# Pharmacophore‐Based Identification and Molecular Characterization of Potent Neprilysin Inhibitors: Biochemical and Therapeutic Implications for Cardiovascular Diseases

**DOI:** 10.1111/cbdd.70247

**Published:** 2026-02-02

**Authors:** Chung‐Ting Kuo, Yi‐Chen Wu, Ji‐Min Li, Tz‐Chuen Ju, Tien‐Sheng Tseng

**Affiliations:** ^1^ Department of Cardiovascular Surgery Ditmanson Medical Foundation Chia‐Yi Christian Hospital Chiayi Taiwan; ^2^ Institute of Molecular Biology, National Chung Hsing University Taichung Taiwan; ^3^ Institute of Precision Medicine, College of Medicine, National Sun Yat‐Sen University Kaohsiung Taiwan; ^4^ Center of Excellence for Metabolic Associated Fatty Liver Disease National Sun Yat‐Sen University Kaohsiung Taiwan; ^5^ International Doctoral Program in Interdisciplinary Innovative Life Science and Technology National Chung Hsing University Taichung Taiwan; ^6^ Doctoral Program in Translational Medicine National Chung Hsing University Taichung Taiwan; ^7^ Doctoral Program in Microbial Genomics National Chung Hsing University and Academia Sinica Taichung Taiwan

**Keywords:** heart failure, LSPR, neprilysin, pharmacophore‐based inhibitor screening, traditional Chinese medicine

## Abstract

Neprilysin (NEP), a zinc‐dependent metalloprotease involved in the degradation of bioactive peptides, represents a validated target for heart failure therapeutics. In this study, a pharmacophore‐based virtual screening approach combined with biochemical and biophysical assays, alongside molecular dynamics (MD) simulations, was employed to identify novel NEP inhibitors. The pharmacophore model **Phar‐A3D2R1** successfully identified pentagalloylglucose (PGG) and tannic acid as potent inhibitors, with IC_50_ values of 17.2 ± 1.5 μM and 10.9 ± 0.7 μM, respectively. Local surface plasmon resonance (LSPR) assays confirmed strong binding affinities (KD = 6.2 ± 0.4 μM for PGG and 5.9 ± 0.5 μM for tannic acid). MD simulations revealed stable ligand–enzyme interactions mediated by hydrogen bonding, hydrophobic contacts, electrostatic interactions, and coordination with the catalytic Zn^2+^ ion. Cytotoxicity assessment in HEK293T cells indicated negligible toxicity. These results validate PGG and tannic acid as promising lead compounds for NEP inhibition and provide a basis for further structure‐based optimization toward cardiovascular therapeutics.

## Graphical abstract

Pharmacophore‐based virtual screening and molecular dynamics identified pentagalloylglucose and tannic acid as potent, non‐toxic neprilysin inhibitors. These natural compounds demonstrated strong binding affinity and stable interactions with the catalytic Zn^2+^ site, offering promising leads for cardiovascular drug development.

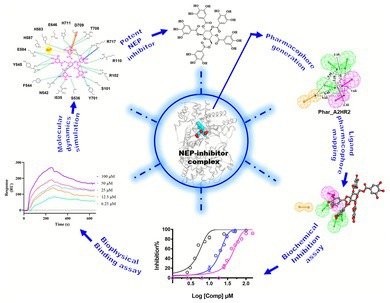

## 1 Introduction

Heart failure (HF) is a complex clinical syndrome defined by the heart's diminished capacity to generate sufficient cardiac output to satisfy the metabolic demands of peripheral tissues, thereby compromising venous return (Kemp and Conte 2012; McMurray et al. 2012). It is typically progressive and insidious, contributing to substantial morbidity and premature mortality. Worldwide, over 22 million individuals are affected by HF, resulting in frequent hospitalizations, impaired quality of life, and a substantial economic burden, with annual costs in the United States alone estimated between $10 and 38 billion (English and Mastrean 1995; Roger et al. 2011). The pathophysiology of HF is multifactorial, with dysregulation of the sympathetic nervous system (SNS) and the renin‐angiotensin‐aldosterone system (RAAS) as central contributors (Volpe et al. 2016). These systems promote progressive myocardial dysfunction, adverse remodeling, and ultimately ventricular failure. Increased SNS activation elevates peripheral vascular resistance, promotes ventricular arrhythmias, and compromises overall cardiac function (Rossi et al. 2017). A critical compensatory mechanism in HF involves the secretion of natriuretic peptides (NPs), including atrial natriuretic peptide (ANP), B‐type natriuretic peptide (BNP), and C‐type natriuretic peptide (CNP) (Bartell and Frishman 2017; Chopra et al. 2013). These peptides counteract maladaptive remodeling by promoting natriuresis, diuresis, vasodilation, and inhibition of cardiac hypertrophy and fibrosis. However, the effectiveness of NPs diminishes as HF progresses, in part due to their enzymatic degradation by neprilysin (NEP) (Schiering et al. 2016). The loss of NP‐mediated cardioprotection underscores the therapeutic relevance of targeting NEP in HF.

NEP (EC 3.4.24.11), also termed neutral endopeptidase, is a zinc‐dependent type II integral membrane metallopeptidase that degrades a broad spectrum of vasoactive peptides, including ANP, BNP, bradykinin, adrenomedullin, and endothelin‐1 (Bayes‐Genis 2015) (Bayes‐Genis et al. 2016; Bellis et al. 2020). By modulating these peptides, NEP directly regulates cardiovascular tone, fluid homeostasis, and renal function. Its activity is highest in the kidney but also present in vascular endothelium, heart tissue, and other organs (Ramanathan and Padmanabhan 2020; Volpe 2014). Dysregulated NEP contributes to HF progression and elevated blood pressure, reinforcing its status as a clinically validated therapeutic target. The pharmacological inhibition of NEP, as exemplified by sacubitril in the combination therapy sacubitril/valsartan (ARNi), has demonstrated improved outcomes in patients with HF with reduced ejection fraction, including reduced hospitalization rates, enhanced cardiac function, and improved renal outcomes compared to ARB monotherapy (Aykan et al. 2019) (Haynes et al. 2020). These observations highlight the potential of NEP‐targeted interventions to restore NP‐mediated cardioprotection and attenuate disease progression.

Despite its therapeutic potential, the repertoire of clinically available NEP inhibitors remains limited. Sacubitril/valsartan represents a milestone in HF therapy, but alternative scaffolds with distinct chemical properties, enhanced safety, or novel modes of interaction are highly desirable. In this context, natural products (NPs) and traditional Chinese medicines (TCMs) offer a rich source of structurally diverse bioactive compounds with favorable pharmacokinetic and safety profiles (Wang et al. 2024; Wainwright et al. 2022). The multivalent polyphenolic scaffolds commonly found in TCMs provide opportunities for engaging NEP through chemically distinct binding modes, complementing synthetic inhibitors and opening avenues for novel lead optimization. Given the central role of NEP in cardiovascular health, TCM‐derived compounds present a promising avenue for discovering next‐generation NEP inhibitors with potential therapeutic benefits in HF and other cardiovascular disorders.

In this study, we hypothesize that polyphenol‐rich compounds derived from traditional Chinese medicine (TCM) constitute a chemically distinct and mechanistically relevant class of neprilysin (NEP) inhibitors, capable of engaging the Zn^2+^‐dependent catalytic pocket through multivalent interactions. This hypothesis is grounded in the unique structural features of TCM‐derived polyphenols, particularly galloyl‐containing scaffolds, which offer multiple hydrogen‐bond donors/acceptors and metal‐coordinating moieties that are largely absent from currently approved synthetic NEP inhibitors. To test this hypothesis, we implemented a structure‐guided discovery workflow integrating pharmacophore‐based virtual screening of a TCM compound library with biochemical inhibition assays, label‐free biophysical binding analysis, cell‐based cytotoxicity evaluation, and molecular dynamics simulations. Using this approach, we identified pentagalloylglucose (PGG, **G5**) and tannic acid (**G10**) as potent NEP inhibitors, exhibiting IC_50_ values of 17.2 ± 1.5 μM and 10.9 ± 0.7 μM, respectively, together with strong binding affinities determined by LSPR analysis (KD = 6.2 ± 0.4 μM for PGG and 5.9 ± 0.5 μM for tannic acid). Molecular dynamics simulations revealed stable NEP‐ligand complexes mediated by hydrogen bonding networks, hydrophobic and electrostatic interactions, and Zn^2+^ coordination, with galloyl group number and spatial arrangement emerging as key determinants of inhibitory potency. Importantly, both compounds showed no apparent cytotoxicity in HEK293T cells. These findings highlight the potential of TCM‐derived NEP inhibitors to restore NP‐mediated cardioprotection, offering a complementary approach to existing therapies for HF patients. Collectively, this work establishes a TCM‐inspired, chemical biology–driven strategy for NEP inhibitor discovery and provides mechanistic insights into polyphenol‐based modulation of NEP activity.

## 2 Materials and Methods

### 2.1 Receptor‐Ligand Pharmacophore Generation and Pharmacophore‐Based Inhibitor Screening (Ligand Pharmacophore Mapping)

To elucidate the critical features required for ligand interactions with target proteins, receptor‐ligand pharmacophore modeling was performed. The pharmacophore model was developed using the crystal structure of the NEP‐sacubitrilat complex (PDB ID: 5JMY). The modeling process was conducted with the receptor‐ligand pharmacophore generation module in Discovery Studio 2021 (Accelrys Software Inc., San Diego, CA, USA). In constructing the pharmacophore model, the NEP structure served as the “Input Receptor,” while the sacubitrilat molecule was designated as the “Input Ligand.” Parameters were configured with “Minimum Features” and “Maximum Features” set to 10 and 30, respectively, and the maximum number of pharmacophores limited to 10. For conformation generation, the “fast method” was utilized in conjunction with the “rigid fitting method.” Default settings were applied to all other parameters. The generated pharmacophore model was subsequently employed for ligand‐pharmacophore mapping to identify potential inhibitors based on the defined pharmacophore features. A total of 1500 compounds from traditional Chinese medicine (TCM) database (https://qatcm.nricm.edu.tw/key‐components/) were screened by fitting them to the pharmacophore model. This screening utilized the “flexible” fitting method, while all other parameters were maintained at their default values.

In comparing a ligand and a pharmacophore, the quality of the mapping is indicated by the fit value. A higher fit value represents a better fit; a perfect mapping of features would result in a fit value equivalent to the sum of the weights of the features in the pharmacophore. The computed fit value depends on two parameters, the weights ***W*** assigned to the pharmacophore features **φ**, and how close the features in the molecule are to the centers of the corresponding location constraints in the pharmacophore. Weights on features indicate the importance of the feature relative to other features. The fit value ***F*** is computed from the sum of all mapped features as:

$$F=\sum^{\text{Mapped Feature}}W\;\left(\emptyset \right)\left[1-\mathrm{SSE}\left(\emptyset \right)\right]$$

where the sum of the square errors **SSE** for all location constraints ***c*** is defined by:

$$\mathrm{SSE}=\sum_{C=1}^{N\text{Constraints}}\left(\frac{D\left(c\right)}{T\left(c\right)}\right)$$

where D is the displacement of the feature from the center of the location constraint, and T is the radius of the location constraint sphere for the feature (tolerance). The hypothesis and conformation are aligned in such a way that the fit is maximized (minimize the weighted sum of square displacements). The above procedure is implemented in *Discovery Studio* and described in the *Discovery Studio 2021 User Guide and Tutorials* (Accelrys Software Inc., San Diego, CA, USA).

### 2.2 Neprilysin Inhibition Assay

A Neprilysin (NEP) inhibition assay was conducted using the Neprilysin Inhibitor Screening Kit (ab284525) purchased from Abcam (Cambridge, UK). The inhibition assay was conducted in accordance with the protocol provided in the kit. The essential steps of the procedure are outlined below. Thiorphan, a well‐characterized neprilysin inhibitor supplied with the Neprilysin Inhibitor Screening Kit (ab284525), was used as a positive control in all enzymatic inhibition assays to validate assay performance and provide a reference for inhibitor potency. The NEP enzyme solution was prepared by reconstituting the protein provided in the kit with 110 μL of NEP assay buffer. The compounds were prepared into the 10 mM stocks and stored at −20°C for future use. A 10 μL of diluted compound was added to a black 96‐well microplate, followed by the addition of 10 μL and 70 μL of NEP assay buffer to each well. The mixture was thoroughly mixed and incubated in the dark at 37°C for 10 min. Following incubation, a substrate mix was prepared by combining 19.5 μL of NEP assay buffer with 0.5 μL of NEP substrate. Afterward, the substrate mix was added to each well. The plate was then placed in an ELISA reader set at 37°C, where fluorescence was measured every 20 min over a 60‐min period, with excitation and emission wavelengths set at 320 nm and 420 nm, respectively. The fluorescence data obtained were used to calculate the inhibition rate using the provided formula.

$$\;\text{Inhibition}\;\left(\%\right)=\frac{\left(\text{Slope}\;\left(\text{control}\right)-\text{Slope}\;\left(\text{tested}\right)\right)}{\text{Slope}\;\left(\text{control}\right)}\times 100\%$$

The IC_50_ of potential inhibitors to NEP was evaluated using the same principles and methodology as described above. Compounds showing inhibitory activity were tested across a range of concentrations to construct dose–response curves and calculate their respective IC_50_ values.

### 2.3 Localized Surface Plasmon Resonance (LSPR)

The binding affinity of inhibitors to NEP was evaluated using localized surface plasmon resonance (LSPR) with an OpenSPR instrument (Nicoya Lifesciences Inc.). The NEP protein (80 μg/mL) was prepared in a Tris‐T buffer (50 mM Tris‐HCl, 150 mM NaCl, 0.005% Tween 20, pH 7.4) and immobilized onto an NTA sensor chip. Inhibitor solutions were prepared by diluting the compounds in Tris‐T buffer supplemented with 0.5% DMSO and 2% BSA, with varying concentrations for analysis. Prior to each measurement, the sensor chip was regenerated using a 10 mM glycine‐HCl buffer at pH 2.2. The resulting data were fitted to a 1:1 binding model using Trace Drawer software to calculate the dissociation constant (KD).

### 2.4 Molecular Dynamics Simulations

To investigate the dynamic interactions of the screened inhibitors (**G5** and **G10**) with NEP, molecular dynamics (MD) simulations were conducted. The structural conformations of the inhibitors **G5** and **G10**, identified via ligand‐pharmacophore (**Phar‐A3D2R1**) mapping, were analyzed through MD simulations. The protein‐ligand complex construction involved acquiring ligand conformations from pharmacophore mapping results and repositioning the ligands within the protein structure to align with the pharmacophore model. Prior to MD simulations, the protein‐inhibitor complexes were refined using the “Prepare Proteins” protocol in Discovery Studio 2021 to resolve structural inconsistencies and ensure the integrity of the NEP–inhibitor complexes. These preparations established a reliable foundation for subsequent computational analyses. Each complex was solvated in an orthorhombic simulation box and parameterized using the CHARMm force field. To simulate physiological conditions, a 0.15 M NaCl ionic strength was employed, incorporating 15,084 water molecules, 50 sodium ions, and 40 chloride ions for the NEP control system. Similar solvation conditions, with minor adjustments for system size, were applied to inhibitor‐bound complexes. Energy minimization of the solvated systems was carried out with 5000 steps of steepest descent followed by 5000 steps of conjugate gradient minimization. The systems were subsequently equilibrated using the “Standard Dynamic Cascade” protocol. A heating phase of 20 ps at 300 K was followed by a 500 ps equilibration under the NVT ensemble. The production phase of the simulations was extended to 100 ns at a constant temperature of 300 K, with snapshots recorded every 500 ps for trajectory analysis. Key structural parameters, including root mean square deviation (RMSD), root mean square fluctuation (RMSF), and radius of gyration (Rg), were analyzed using the “Analyze Trajectory” protocol in Discovery Studio 2021. These analyses, referenced against the initial structure, provided quantitative insights into conformational dynamics and the stability of the NEP–inhibitor complexes over time.

### 2.5 Cytotoxicity Assay

Human embryonic kidney (HEK) 293T cells were cultured in Dulbecco's Modified Eagle Medium (DMEM) supplemented with 10% fetal bovine serum (FBS), 100 U/mL penicillin, and 0.1 mg/mL streptomycin and maintained at 37°C in a humidified atmosphere containing 5% CO_2_. For the cytotoxicity assay, cells were seeded at a density of 5000 cells per well in 96‐well plates and incubated for 24 h to allow for proper attachment. Test compounds (**G5** and **G10**) were freshly prepared in culture medium immediately before use. Due to solubility considerations, high concentrations of **G5** and **G10** were dissolved in 0.5% DMSO, whereas low and standard concentrations were directly soluble in 1× PBS. Accordingly, 0.5% DMSO was used as the vehicle control for high‐concentration groups, while 1× PBS served as the control for low‐ and standard‐concentration groups. This approach ensures that any observed cytotoxic effects are attributable to the compounds themselves rather than differences in solvent exposure. After 24 h of incubation with the compounds, the culture medium was discarded, and the cells were washed once with 1× PBS. Cell viability was assessed using crystal violet staining: 100 μL of 0.1% crystal violet solution was added to each well and incubated at room temperature for 15 min. Excess dye was removed, wells were rinsed with distilled water, and the bound dye was solubilized with 100 μL of 33% acetic acid. Cell viability was quantified by calculating the percentage of viable cells relative to untreated control wells, according to the following formula: Cell Viability (%) = (OD_570_ of treated wells/OD_570_ of control wells) × 100.

## 3 Results

### 3.1 Receptor‐Ligand Pharmacophore Generation

**FIGURE 1:**
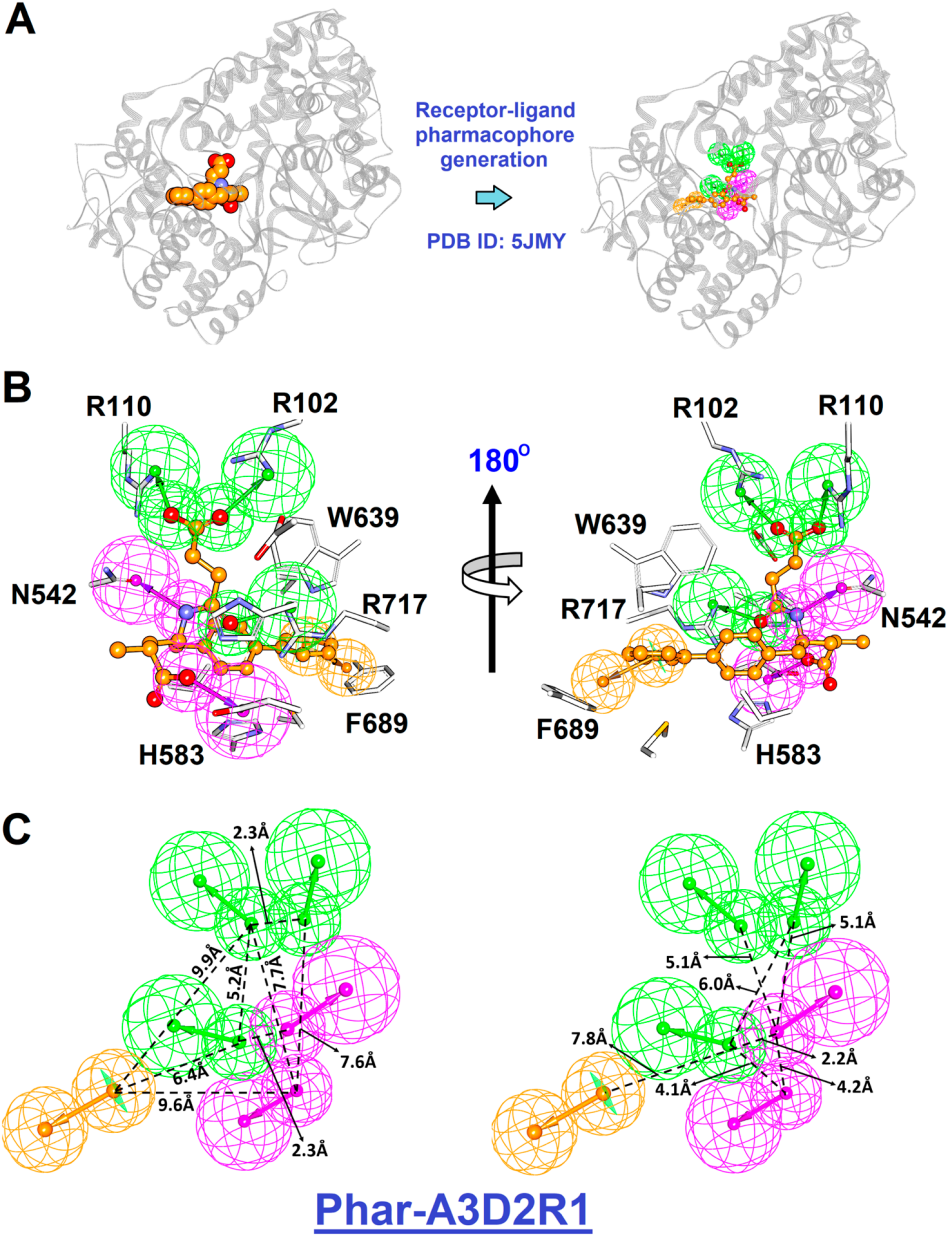
Receptor‐ligand‐pharmacophore generation based on the complex structure of NEP‐sacubitrilat. (A) The complex structure of NEP‐sacubitrilat (PDB ID: 5JMY). The NEP is presented in ribbon (colored in gray); sacubitrilat is shown in spheres (colored in orange). (B) Generated pharmacophore features and structure of the NEP‐sacubitrilat. The interactive residues of NEP are shown as sticks (white) and labeled. The sacubitrilat is presented in balls‐and‐sticks (orange). (C) Features at a specific distance correspond to the pharmacophore model **Phar‐A3D2R1**. Pharmacophore features are color‐coded as follows: Hydrogen‐bond acceptor, green; hydrogen‐bond donor, magenta; ring aromatic feature, orange.

To efficiently identify potent inhibitors of NEP, it is essential to analyze the functional features that mediate interactions between NEP and its known inhibitors. Pharmacophore modeling has proven to be a vital tool for identifying and characterizing key ligand elements, enabling precise receptor binding. This approach translates protein properties into ligand‐specific features, providing a framework to elucidate the critical attributes governing inhibitor‐target interactions (Lu et al. 2018; Pirhadi et al. 2013; Yang 2010). The receptor–ligand pharmacophore model was generated based on the reported crystal structure of the human NEP–sacubitrilat complex, as sacubitrilat represents a clinically validated neprilysin (NEP) inhibitor with a well‐defined binding mode suitable for structure‐guided feature extraction. The crystal structure of the NEP–sacubitrilat complex (PDB ID: 5JMY) provides high‐resolution structural information on key interactions within the NEP catalytic pocket, including Zn^2+^ coordination, hydrogen‐bonding networks, and subsite occupation, and therefore served as a reliable template for pharmacophore modeling (Figure 1A). A receptor‐ligand pharmacophore generation process was conducted, with NEP defined as the receptor and sacubitrilat as the ligand. This process culminated in the development of the pharmacophore model, **Phar‐A3D2R1**, which encapsulates the essential features for NEP‐inhibitor interactions (Figure 1B). **Phar‐A3D2R1** comprises three hydrogen‐bond acceptors (green spheres), two hydrogen‐bond donors (magenta spheres), and one aromatic ring feature (orange sphere) (Figure 1C). These features collectively represent the critical elements required for sacubitrilat binding to NEP. The model offers a comprehensive representation of the necessary attributes for NEP binding, providing a valuable tool for understanding NEP‐inhibitor interactions and aiding the rational design of novel inhibitors.

### 3.2 Pharmacophore‐Based Inhibitor Screening

**FIGURE 2:**
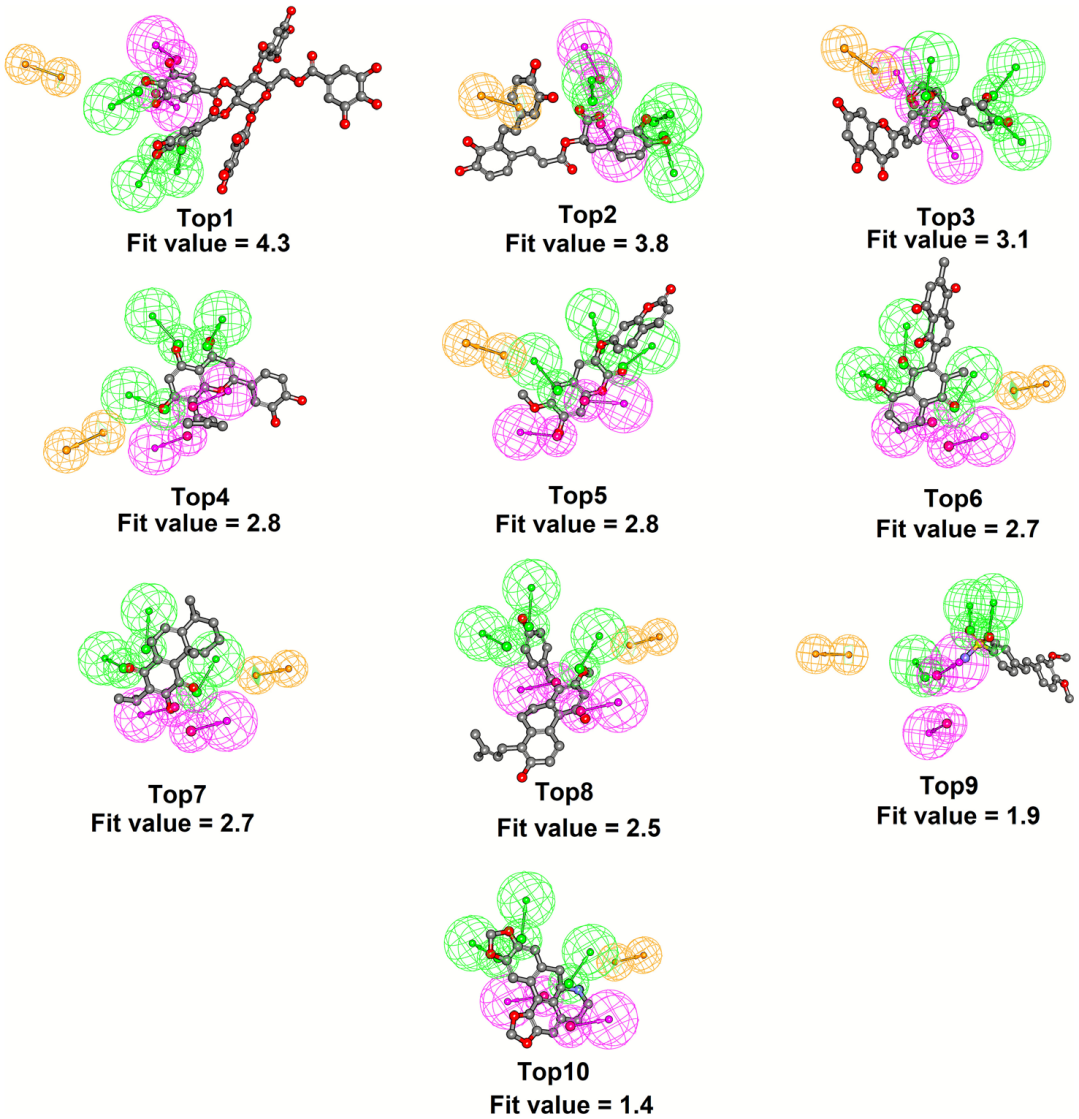
Pharmacophore‐based inhibitor screening. The results of ligand pharmacophore (**Phar‐A3D2R1**) mapping of top 10 hits screened from TCM database.

High‐throughput inhibitor screening via pharmacophore modeling necessitates the careful selection of an appropriate pharmacophore scaffold to ensure effective ligand‐pharmacophore alignment. In this study, we utilized the pharmacophore model **Phar‐A3D2R1** to conduct a systematic screening of a library consisting of 1500 compounds derived from the traditional Chinese medicine (TCM) database. The ligand‐pharmacophore mapping involved aligning the three‐dimensional structures of each compound to the key pharmacophore features of **Phar‐A3D2R1** (Figure 1C). This alignment enabled an accurate assessment of how well each compound fit the pharmacophore. Fit scores were calculated to quantify the degree of compatibility, with higher values indicating stronger and more favorable interactions between the ligand and the pharmacophore. These scores provided a basis for ranking the compounds, allowing for the identification of the most promising candidates. Based on these rankings, the top 10 compounds were selected for further experimental validation (Figure 2). These top candidates displayed high fit scores, indicating their strong potential for effective interaction with NEP. The chemical structures of these compounds are shown in Figure S1.

### 3.3 NEP Inhibitory Activity

**FIGURE 3:**
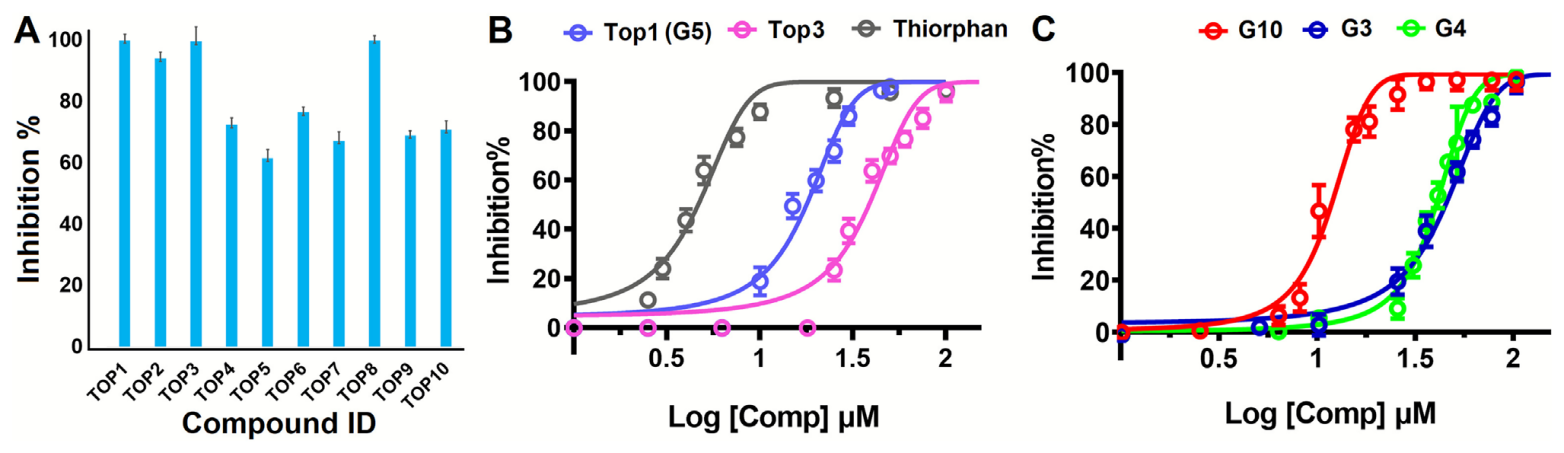
Inhibitory potencies of identified inhibitors against NEP. (A) The inhibitory effects of compounds were tested at 100 μM against NEP. (B) Dose‐dependent inhibition curves of **Top1 (G5)** and **Top3** against NEP. (C) Dose‐dependent inhibition curves of **G3**, **G4**, and **G10** against NEP.

**FIGURE 4:**
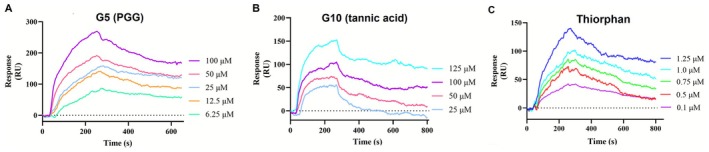
SPR sensorgrams of G5 (PGG) and G10 (tannic acid) binding with NEP. Binding affinity of (A) **G5** (PGG) to NEP (KD = 6.2 ± 0.4 μM), (B) **G10** (tannic acid) to NEP (KD = 5.9 ± 0.5 μM), (C) Thiorphan to NEP (KD = 0.15 ± 0.08 μM).

A fluorescence‐based inhibition assay was employed to evaluate the inhibitory ability of the screened top 10 hits. Initially, the NEP inhibitory effects of compounds were assessed at 100 μM. The result of preliminary screening showed that 4 natural products (Top1, Top2, Top3, and Top8) exerted > 94% inhibition against NEP, respectively (Figure 3A and Figure S1). Subsequently, these inhibitors were subjected to further inhibition assessments at various concentrations to determine their IC_50_ values. The results demonstrated that candidates, Top1 and Top3, exhibited a dose‐dependent inhibition against NEP (Figure 3B). The IC_50_ values for Top1 and Top3 were determined to be 17.2 ± 1.5 and 37.8 ± 2.1 μM, individually. While the IC_50_ values of candidates, Top2 and Top8, were not available due to serious aggregations of NEP upon addition of high concentration of compounds. As expected, thiorphan exhibited potent NEP inhibition (IC_50_ = 0.64 ± 0.07 μM), consistent with its established role as a reference NEP inhibitor, thereby validating the reliability of the enzymatic assay. Notably, Top1 (**G5**) demonstrated the apparent inhibitory potency against NEP, leading to subsequent experimental assessments of inhibitory efficacy in its structural analogs and derivatives. About 5 analogs (**G0**, **G1**, **G3**, **G4**, and **G10**) were selected for further assessment of inhibitory potential (Figure 4). Preliminary inhibition assays at 100 μM compound concentration demonstrated that **G3**, **G4**, and **G10** showed apparent inhibitory activity (data not shown), meriting further investigation through IC_50_ assays to quantify their efficacy. Consequently, the IC_50_ values of **G3**, **G4**, and **G10** were determined to be 42.6 ± 2.2, 38.6 ± 1.8, and 10.9 ± 0.7 μM, respectively (Figure 3C).

### 3.4 The Binding Affinities of **G10** and **G5** Towards NEP

The inhibitory assay revealed that natural products, **G5** and **G10**, were potent inhibitors targeting NEP. To further confirm their interactions and binding to NEP, we conducted affinity analysis using local surface plasmon resonance (LSPR). This technique enabled us to assess the interaction dynamics by measuring the association (K_on_) and dissociation (K_off_) rates between NEP and compound at various concentrations, leading to the determination of the equilibrium dissociation constant (KD). During the experiments, **G5** was tested at concentrations of 6.25, 12.5, 25, 50, and 100 μM against NEP. The sensorgrams revealed a fast association and slow dissociation binding pattern of **G5** with NEP, resulting in a KD value of 6.2 ± 0.4 μM (Figure 4A). Similarly, the binding of **G10** to NEP was investigated at concentrations of 25, 20, 100, and 200 μM.

The sensorgrams indicated a binding pattern with rapid association and slow dissociation phases. The KD was 5.9 ± 0.5 μM (Figure 4B), highlighting comparable binding affinity between **G10** and NEP. The binding affinity of thiorphan to NEP was determined to be 0.15 ± 0.08 μM (Figure 4C).

### 3.5 The Cytotoxicity of G5 and G10

**FIGURE 5:**
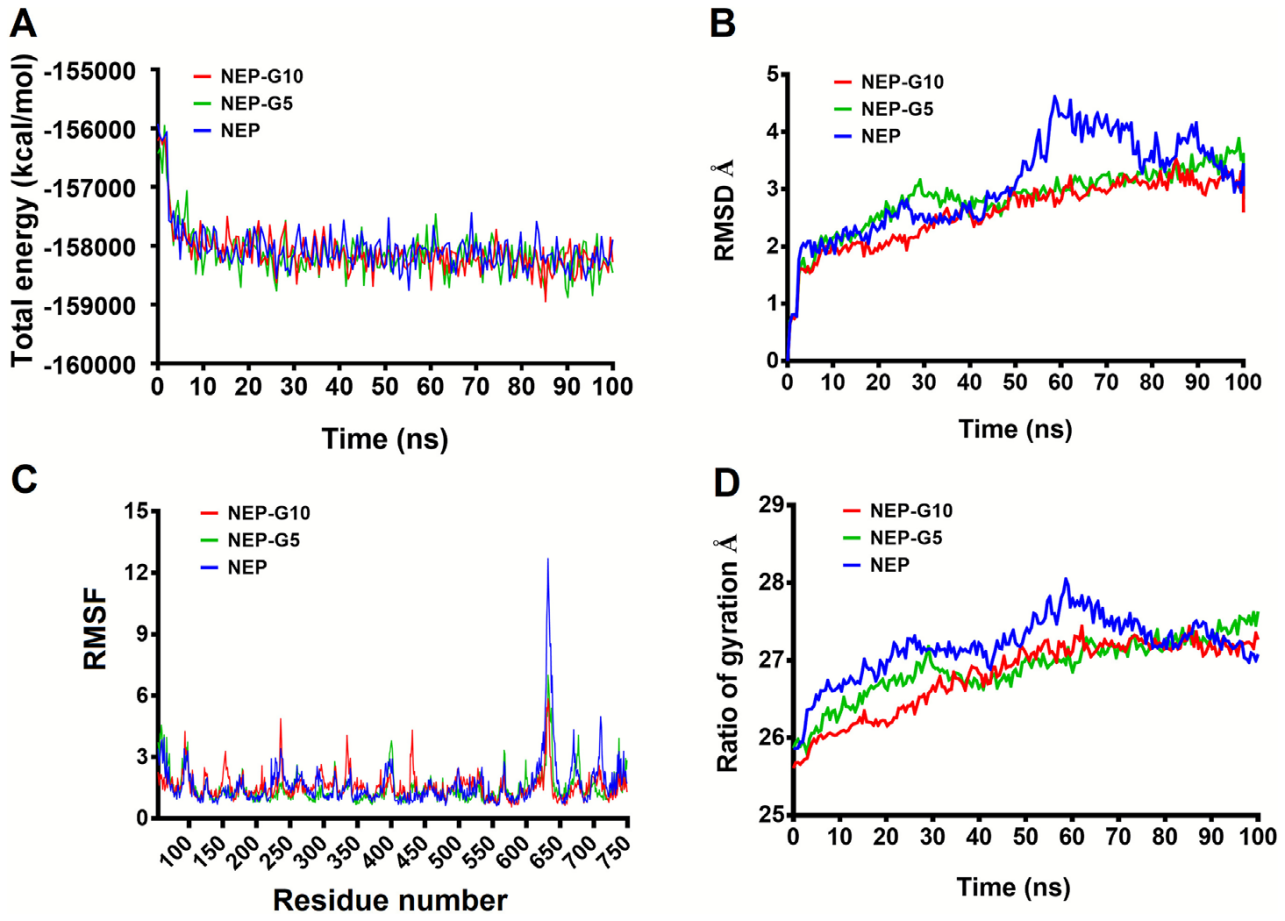
Stability parameters obtained from the MD simulation analysis and binding free energy. (A) The total energy of NEP alone and in complex with inhibitors as a function of simulation time. (B) RMSD of protein‐ligand complexes as a function of simulation time. (C) RMSF values of the NEP residues. (D) Rg values as a function of simulation time.

**FIGURE 6:**
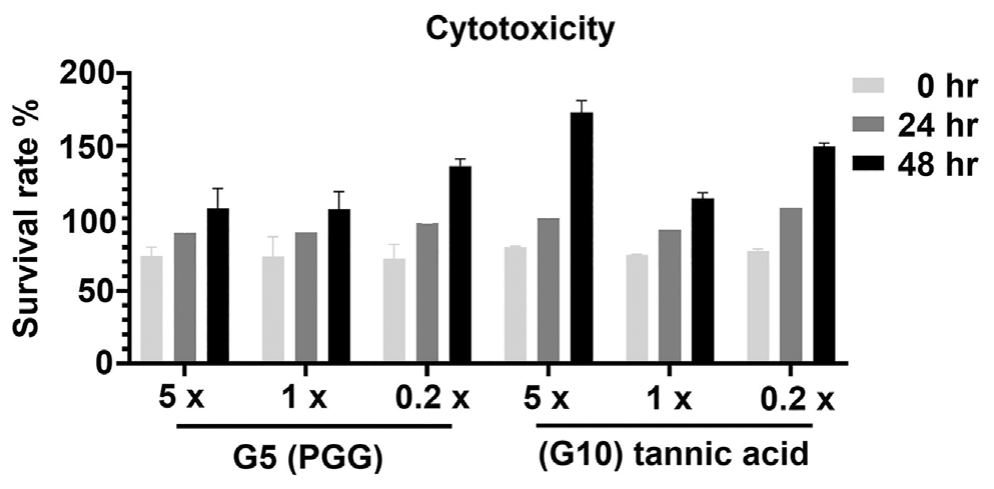
Cell viability of HEK293T under PGG and tannic acid treatments.

To assess the cytotoxicity of **G10** and **G5**, HEK293T cells were treated with a range of concentrations: high (5 folds), standard (1 folds), and low (0.2 folds) relative to the IC_50_ values. Cell viability was monitored to determine the effect of each concentration on cell growth, with 0.5% DMSO serving as the control for the high concentration and 1 × PBS for the standard and low concentrations. The results showed that the cell viabilities of the conditions treated with **G10** and **G5** showed no significant change compared to that of the mock control (Figure 5). These results indicated that **G10** and **G5** have no apparent cytotoxicity to mammalian cells (Figure 6).

### 3.6 Molecular Dynamics Simulations Revealed the Mode of Actions of G5 and G10

To gain a detailed understanding of the atomic‐level interactions between NEP and the identified inhibitors, the molecular dynamics (MD) simulations were performed. MD simulations were performed to assess the binding stability of the NEP‐inhibitor complexes (**G5** and **G10**) over 100 ns, with key structural parameters analyzed, including root mean square deviation (RMSD), root mean square fluctuation (RMSF), and radius of gyration (Rg). The total energies of NEP alone and in complex with inhibitors as a function of simulation times were shown in Figure 5A. The average RMSD for NEP alone was 3.05 Å, compared to 2.82 Å for the NEP‐**G5** complex and 2.59 Å for NEP‐G10 (Figure 5B), indicating enhanced structural stability in the inhibitor‐bound states. RMSF analysis revealed similar fluctuation patterns across NEP, NEP‐**G5**, and NEP‐**G10** (Figure 5C), suggesting that inhibitor binding enhanced the overall stability and altered dynamic behavior of NEP residues. The Rg provided further insights into the compactness and stability of the complexes. NEP alone exhibited Rg values fluctuating between 25.86 and 28.05 Å, reflecting a relatively unstable conformation (Figure 5D). In contrast, the NEP‐**G5** complex showed more consistent Rg fluctuations between 25.79 and 27.62 Å, while the NEP‐**G10** complex demonstrated the most stable Rg values, fluctuating between 25.60 and 27.43 Å. These findings indicate that both **G5** and **G10** form thermodynamically stable complexes with NEP, with **G10** exhibiting slightly enhanced stability.

### 3.7 Analyses of Molecular Interactions of G5 and G10 With NEP

**FIGURE 7:**
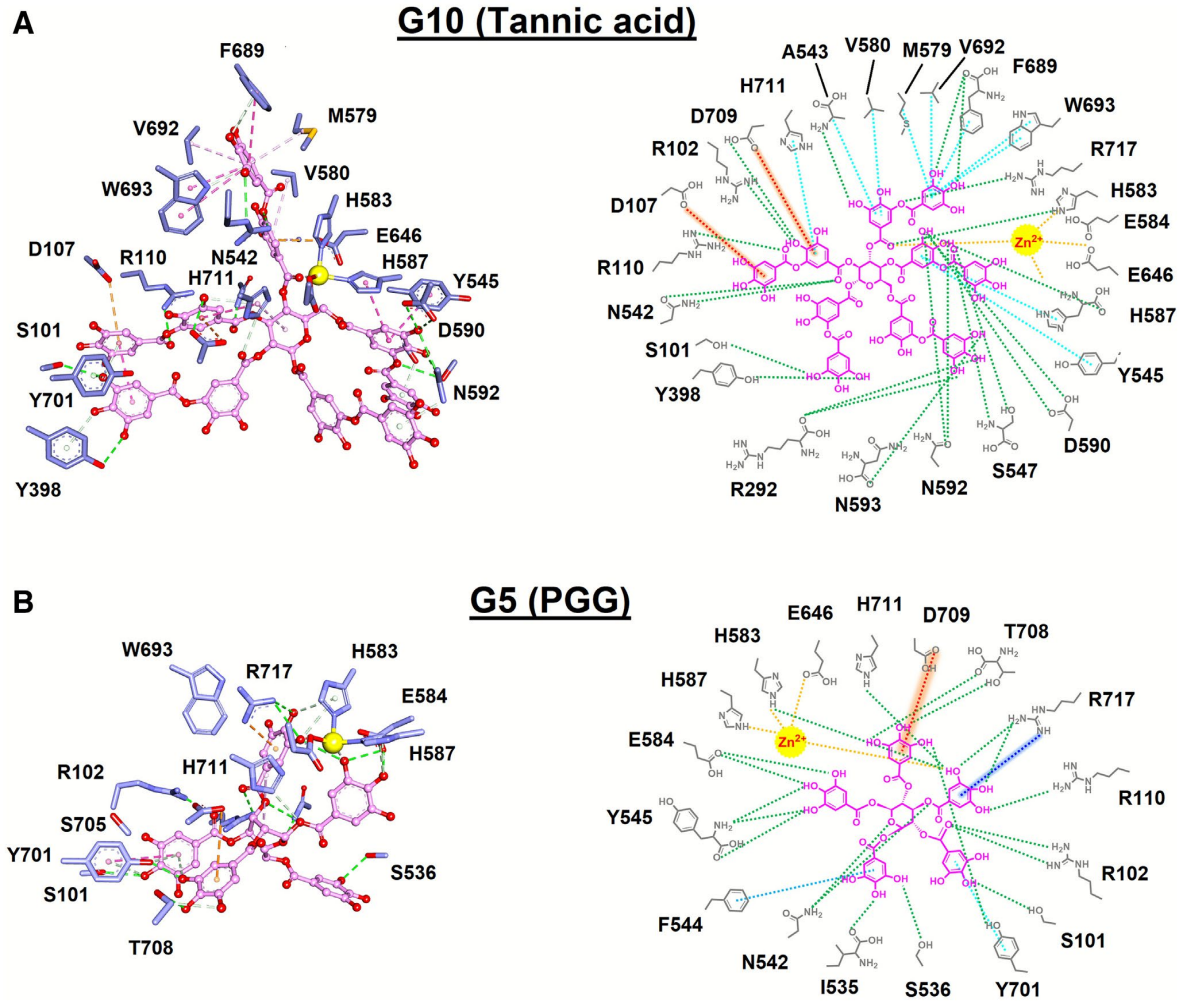
Molecular interactions of PGG and tannic acid with NEP. Molecular interactions of (A) tannic acid and (B) PGG with NEP from MD simulations at the 100 ns time point. In all three‐dimensional structural plots, tannic acid and PGG are presented as ball‐and‐sticks (pink); interactive residues of NEP are displayed as white sticks (light blue) and labeled. In all two‐dimensional schematic plots, the chemical structures of the inhibitors are colored in pink, the interactive residues of NEP are labeled, and the cyan, green, yellow, red and blue dashed lines indicate hydrophobic interactions, hydrogen bonding, coordination with Zn^2+^, anion‐pi interaction, and cation‐pi interaction, respectively.

To better understand the molecular interactions between the identified inhibitors, **G10** and **G5**, with NEP, structural analyses were performed using the non‐bond interaction analysis module in Discovery Studio 2021. The analysis of **G5** and **G10** interactions with NEP revealed comprehensive binding within the active site, with each inhibitor displaying unique interaction profiles involving key NEP residues (Figure 7). In the three‐dimensional structural representations, **G5** and **G10** were depicted as pink ball‐and‐stick structures, interacting with key residues of NEP, visualized as light blue sticks and explicitly labeled (Figure 7). Structurally, **G5** formed hydrogen bonds with 13 key residues of NEP, while also engaging in hydrophobic interactions with Y701 and F544 (Figure 7). Similarly, **G10** established more hydrogen bonds with 16 key residues of NEP and hydrophobically contacted A543, Y545, M579, V580, H587, V692, W693, F689, and H711 (Figure 7). The schematic representations highlight the variety of interactions: hydrogen bonds (green dash), hydrophobic interactions (cyan dash lines), anion‐pi interactions (red dash lines), cation‐pi interactions (blue dash lines), and (yellow dash lines) coordination with Zn^2+^ ion. These results emphasize the role of hydrogen bonding and hydrophobic interactions in stabilizing both inhibitors, with **G10** displaying a denser network of hydrogen bonds, while **G5** exhibits stronger hydrophobic and electrostatic contacts. This analysis highlights the critical interactions that stabilize the inhibitors within the active site of NEP, offering structural insights into their potential as effective NEP inhibitors.

## 4 Discussion

Heart failure is a critical global health issue, affecting over 26 million individuals worldwide (Wei et al. 2024). In the United States and Europe, over one million patients are hospitalized annually (Beezer et al. 2022; Ziaeian and Fonarow 2016). Its rising prevalence is driven by aging and unhealthy diets combined with low physical activity (Grillo et al. 2019; Petrie et al. 2018; Reuter et al. 2019). Elevated circulating natriuretic peptides (NPs) and increased neprilysin (NEP) expression serve as key biomarkers (Castiglione et al. 2022). NPs regulate blood pressure via receptor‐mediated vasodilation but are degraded by NEP, highlighting NEP inhibition as a therapeutic strategy (Diez 2017; Ito et al. 2015; Mangiafico et al. 2013). Currently, most NEP inhibitors are chemically synthesized (Bevan et al. 1992; Kawanami et al. 2020; Mascarello et al. 2021; Matiadis et al. 2021; McKinnell et al. 2019; Mizerska‐Kowalska et al. 2019; Sankhe et al. 2021) and are associated with significant adverse effects (AlAnazi et al. 2023; Ali et al. 2024; McMurray et al. 2014; Singh et al. 2017; Wewer Albrechtsen et al. 2022). Plant‐derived compounds show therapeutic potential against multiple diseases (Asafo‐Agyei et al. 2023; Chhabria et al. 2022; Kumar et al. 2023; Pagliaro et al. 2015; Rudzinska et al. 2023; Tsai et al. 2022). Also, computer‐aided drug design (CADD), integrating molecular docking and pharmacophore modeling, provides a strategy to identify bioactive compounds (Kaur and Khatik 2021; Talevi 2018).

In this study, a pharmacophore model based on the NEP–sacubitrilat crystal structure (PDB ID: 5JMY) was successfully constructed to guide the identification of potential NEP inhibitors. The resulting **Phar‐A3D2R1** model, comprising three hydrogen‐bond acceptors, two hydrogen‐bond donors, and one aromatic ring, effectively captured the essential ligand‐binding interactions and enabled virtual screening of candidate compounds. Among the top‐ranked hits, **G5** (PGG) demonstrated notable NEP inhibition (IC_50_ = 17.2 ± 1.5 μM), while its analogs **G3**, **G4**, and **G10** exhibited over 90% inhibition at 100 μM. Dose–response assays further revealed that **G10** (tannic acid), with 10 galloyl groups on a glucose/polyol core, showed the strongest activity (IC_50_ = 10.9 ± 0.7 μM), whereas **G4** and **G3** displayed moderate to low potency. These findings suggest that both the number and spatial arrangement of galloyl groups are critical determinants of NEP inhibition, emphasizing the importance of multivalent polyphenolic interactions in stabilizing the enzyme‐inhibitor complex. Thiorphan, a classical small‐molecule NEP inhibitor, was used as a reference due to its established enzymatic activity in vitro. Although PGG and tannic acid are less potent than thiorphan, their multivalent polyphenolic scaffolds engage NEP via distinct interactions, supporting their potential as alternative lead compounds for further optimization.

Additionally, LSPR analysis indicated that **G5** and **G10** exhibit comparable binding profiles with NEP, with KD values of 6.2 ± 0.4 μM and 5.9 ± 0.5 μM, respectively, confirming effective interactions between these polyphenolic compounds and the enzyme. This similarity in binding affinities highlights their potential as lead compounds for further optimization. Molecular dynamics simulations provided additional insights into the binding dynamics and structural consequences of inhibitor engagement. Both NEP‐**G5** and NEP‐**G10** complexes showed reduced RMSD values compared to NEP alone, suggesting enhanced overall stability upon inhibitor binding. Although RMSF profiles indicated minimal local flexibility changes, analysis of the radius of gyration (Rg) revealed that both inhibitors reduced the extent of global structural fluctuations. Notably, NEP‐**G10** displayed the narrowest Rg range, implying the formation of the most thermodynamically stable complex. These results collectively suggest that, while both **G5** and **G10** stabilize NEP, **G10** may serve as a superior stabilizer, potentially improving substrate‐binding efficiency and overall enzymatic regulation. Such insights provide a mechanistic basis for prioritizing **G10** in future optimization efforts as a NEP‐targeted therapeutic.

Moreover, molecular dynamics simulations and non‐bonded interaction analyses revealed strong and stable interactions between NEP and both **G5** (PGG) and **G10** (tannic acid) (Figure 7). Similar to the reference human NEP inhibitor sacubitrilat, both compounds were stabilized by a combination of hydrogen bonding, hydrophobic interactions, electrostatic forces, and Zn^2+^ coordination, indicating a conserved inhibitory binding mode within the NEP active site. Sacubitrilat forms hydrogen bonds with key residues including R102, R110, N542, and R717, together with hydrophobic interactions involving V580, H583, F689, V692, and W693, as well as π–sulfur (M579) and cation–π (R717) interactions (Figure S2). Coordination with the catalytic Zn^2+^ ion further stabilizes the NEP‐sacubitrilat complex. In comparison, **G5** establishes an expanded hydrogen‐bonding network with residues S101, R102, R110, I535, S536, N542, Y545, H583, E584, Y701, T708, H711, and R717. Additional stabilization arises from hydrophobic interactions with Y701 and F544, as well as cation–π (R717) and anion–π (D709) interactions, complemented by Zn^2+^ coordination. While **G5** shares several conserved interaction hotspots with sacubitrilat, such as R102, R110, N542, H583, and R717, its broader hydrogen‐bonding pattern suggests enhanced surface complementarity within the active site. **G10** exhibits the most extensive interaction profile among the three inhibitors, forming hydrogen bonds with S101, R102, R110, R292, Y398, N542, A543, S547, H583, H587, D590, N592, N593, F689, D709, and R717. Hydrophobic contacts with A543, Y545, M579, V580, H587, V692, W693, F689, and H711, together with electrostatic stabilization at D709 and D107, further anchor **G10** within the binding pocket. Notably, Zn^2+^ coordination mediated by H583 plays a central role in stabilizing the NEP‐**G10** complex, resembling but extending beyond the metal‐binding interactions observed for sacubitrilat. Collectively, while sacubitrilat represents a well‐characterized benchmark inhibitor with a defined interaction core, **G5** and especially **G10** demonstrate more extensive hydrogen‐bonding and non‐covalent interaction networks. These findings suggest that **G5** and **G10** may achieve strong NEP inhibition through cooperative interactions involving hydrogen bonding, hydrophobic and electrostatic forces, and metal coordination. Such features provide a promising basis for further structural optimization aimed at improving inhibitory potency and selectivity for therapeutic targeting of NEP.

The cytotoxicity of **G5** (PGG) and **G10** (tannic acid) was evaluated in HEK293T cells (Figure 6). Neither compound showed noticeable cytotoxicity, confirming their safety. High concentrations of tannic acid promoted HEK293T proliferation after 48 h, consistent with previous reports (Chen et al. 2021; Perumal et al. 2019). PGG, a polyphenolic compound found in fruits such as black walnut (Vu et al. 2020), pomegranate (Feng et al. 2019), and mango (Mahmoud et al. 2022), consists of five ester‐linked gallic acid moieties on a glucose core, enhancing its antioxidant potency relative to gallic acid (IC_50_ = 7.1 ± 0.26 μM vs. 12.1 ± 0.16 μM) (Shaikh et al. 2016). PGG exhibits broad‐spectrum antibacterial activity, including against antibiotic‐resistant *Staphylococcus aureus* (Jiamboonsri et al. 2011), antiviral effects against HSV‐1 and influenza A virus (Haid et al. 2015; Jin et al. 2016), anticancer activity by inducing apoptosis and inhibiting metastasis (Deiab et al. 2015; Li et al. 2011; Zhao et al. 2014), and anti‐inflammatory effects via neutrophil recruitment modulation and L‐selectin inhibition (Jang et al. 2013; Kiss et al. 2013; Zhao et al. 2015). In this study, PGG was identified as a potent natural NEP inhibitor. Biochemical assays revealed strong inhibition (IC_50_ of 17.2 ± 1.5 μM), with LSPR showing a KD of 6.2 ± 0.4 μM. Molecular docking and dynamics simulations indicated stable binding through hydrogen bonds, hydrophobic and electrostatic interactions, and coordination with the catalytic Zn^2+^ ion, enhancing inhibitory efficacy. These results highlight PGG as a versatile lead compound for NEP‐targeted therapy. Its broad pharmacological profile suggests potential applications in cardiovascular disease, oncology, infectious diseases, and metabolic disorders. Future studies on structural optimization and mechanistic exploration are needed to realize PGG's therapeutic potential.

**FIGURE 8:**
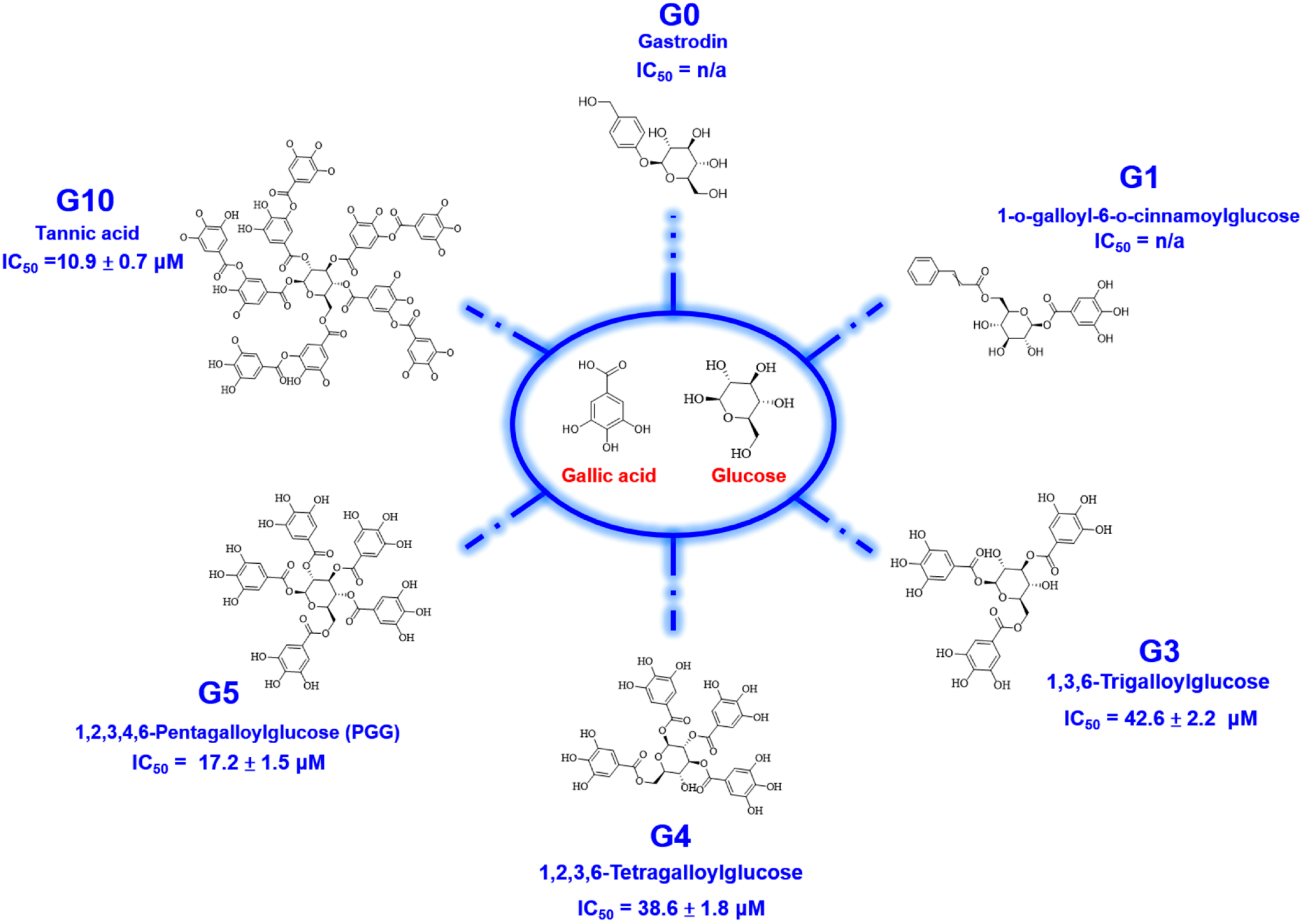
Structure and inhibitory activity of galloylglucose derivatives. The central structure represents gallic acid and glucose, the core components of the displayed compounds. Surrounding this central structure are various galloylglucose derivatives (**G0a**, **G0b**, **G1**, **G3**, **G4**, **G5**, and **G10**) with their respective chemical structures and half‐maximal inhibitory concentration (IC_50_) values, where applicable. Compounds with no available IC₅₀ values are marked as “n/a.” Notably, tannic acid (**G10**) shows the strongest inhibitory effect with an IC₅₀ of 10.9 ± 0.7 μM, followed by PGG (**G5**) with an IC₅₀ of 17.2 ± 1.5 μM.

**G10** (tannic acid), a plant‐derived polyphenol present in red wine (Chu et al. 2015), coffee (Savolainen 1992), grapes (Karuppagounder et al. 2015), and chocolate (Chung et al. 1998; King and Young 1999), exhibits various therapeutic properties. Supplementation at 0.2%–1.0% reduces post‐weaning diarrhea in livestock, indicating potential in veterinary medicine (Song et al. 2021; Yu et al. 2020). Combined with aluminum potassium sulfate, it aids in managing internal hemorrhoids (Tomiki et al. 2019). Tannic acid also shows anticancer activity, inhibiting liver, breast, and colorectal tumor growth via antioxidant activity and modulation of cancer cell signaling and gene expression (Athar et al. 1989; Horikawa et al. 1994; Wu et al. 2004). Additionally, tannic acid exhibits antimicrobial activity against MRSA (Akiyama et al. 2001; Dong et al. 2018; Kirmusaoglu 2019) and antiviral activity against influenza A and HSV‐2, particularly when combined with nano‐silver particles (Orlowski et al. 2018, 2014). In our study, **G10** emerged as a potent NEP inhibitor, with an IC_50_ of 10.9 ± 0.7 μM (Figure 8) and KD of 5.9 ± 0.5 μM (Figure 4). Molecular interactions, including hydrogen bonding, hydrophobic, and electrostatic forces, stabilized the NEP‐tannic acid complex (Figure 7). Beyond NEP inhibition, tannic acid's broad pharmacological profile—antioxidant, anti‐inflammatory, antimicrobial, and anticancer—supports its potential in oncology, infectious diseases, and gastrointestinal health. Despite solubility and bioavailability challenges, its strong activity and binding affinity highlight tannic acid as a promising lead for NEP‐targeted therapies. Collectively, these findings emphasize the significance of PGG and tannic acid as bioactive compounds with potential for developing NEP‐targeted therapeutic agents.

## 5 Conclusion

In this study, we identified pentagalloylglucose (PGG) and tannic acid as potent NEP inhibitors using a pharmacophore‐based inhibitor screening complemented by biochemical and biophysical analyses. Both compounds exhibited strong inhibitory activity (IC_50_: 17.2 ± 1.5 μM for PGG and 10.9 ± 0.7 μM for tannic acid) and high binding affinities (KD: 6.2 ± 0.4 μM and 5.9 ± 0.5 μM, respectively), as determined through local surface plasmon resonance analysis. Molecular docking and molecular dynamics simulations revealed stable and specific interactions with NEP, mediated by hydrogen bonding, hydrophobic interactions, electrostatic forces, and Zn^2+^ ion coordination. Importantly, cytotoxicity assays confirmed the nontoxic nature of these compounds in HEK293T cells, underscoring their therapeutic potential. These findings establish PGG and tannic acid as promising NEP inhibitors, offering a strong foundation for further optimization and preclinical studies to advance the development of targeted therapies for heart failure.

## Author Contributions

**Chung‐Ting Kuo:** investigation, validation, data curation. **Yi‐Chen Wu:** investigation, validation, data curation. **Ji‐Min Li:** data curation, validation. **Tz‐Chuen Ju:** data curation, validation. **Tien‐Sheng Tseng:** conceptualization, investigation, methodology, data curation, writing – original draft, writing – review and editing, supervision, funding acquisition.

## Funding

This study was supported by the National Science and Technology Council, Taiwan (NSTC 112‐2320‐B‐005‐010‐MY3).

## Conflicts of Interest

The authors declare no conflicts of interest.

## Supporting information


**Figure S1:** The chemical structures of top 10 ranked hits identified from TCM database.
**Figure S2:** Molecular interactions between human neprilysin (NEP) and sacubitrilat. Sacubitrilat is shown in ball‐and‐stick representation (gray), while key NEP residues are depicted as sticks. Hydrogen bonds are indicated by green dashed lines, hydrophobic interactions by magenta dashed lines, and π–sulfur interactions by yellow dashed lines. Cation–π interactions involving R717 are also observed. Residues contributing to ligand stabilization include R102, R110, N542, R717, V580, H583, F689, V692, and W693. Coordination with the catalytic Zn^2^⁺ ion (yellow sphere), mediated by H583 and H587, further anchors sacubitrilat within the NEP active site, highlighting key interaction hotspots characteristic of known human NEP inhibitors.

## Data Availability

The data that support the findings of this study are available on request from the corresponding author. The data are not publicly available due to privacy or ethical restrictions.
