# Supplementary material for: Pharmacophore‐Based Identification and Molecular Characterization of Potent Neprilysin Inhibitors: Biochemical and Therapeutic Implications for Cardiovascular Diseases
Source: Chem Biol Drug Des. 2026 Feb 2;107(2):e70247. doi: 10.1111/cbdd.70247 (PMC12893245; doi:10.1111/cbdd.70247)
Supplement: Supplementary file 1 — Figure S1: The chemical structures of top 10 ranked hits identified from TCM database. Figure S2: Molecular interactions between human neprilysin (NEP) and sacubitrilat. Sacubitrilat is shown in ball‐and‐stick representation (gray), while key NEP residues are depicted as sticks. Hydrogen bonds are indicated by green dashed lines, hydrophobic interactions by magenta dashed lines, and π–sulfur interactions by yellow dashed lines. Cation–π interactions involving R717 are also observed. Residues contributing to ligand stabilization include R102, R110, N542, R717, V580, H583, F689, V692, and W693. Coordination with the catalytic Zn2⁺ ion (yellow sphere), mediated by H583 and H587, further anchors sacubitrilat within the NEP active site, highlighting key interaction hotspots characteristic of known human NEP inhibitors. [file CBDD-107-e70247-s001.docx]

**Pharmacophore-Based Identification and Molecular Characterization of Potent Neprilysin Inhibitors: Biochemical and Therapeutic Implications for Cardiovascular Diseases**

**Chung-Ting Kuo^a#^, Yi-Chen Wu****^b#^, Ji-Min Li^e, f^, Tz-Chuen Ju^b, d, g^ and Tien-Sheng Tseng^b, c, d*^**

^a^Department of Cardiovascular Surgery, Ditmanson Medical Foundation Chia-Yi Christian Hospital, Chiayi, Taiwan.

^b^Institute of Molecular Biology, National Chung Hsing University, Taichung, Taiwan.

^c^Doctoral Program in Microbial Genomics, National Chung Hsing University and Academia Sinica, Taiwan.

^d^International Doctoral Program in Interdisciplinary Innovative Life Science and Technology, National Chung Hsing University, Taichung, Taiwan.

^e^Institute of Precision Medicine, College of Medicine, National Sun Yat-sen University, Kaohsiung, 80424, Taiwan.

^f^Center of Excellence for Metabolic Associated Fatty Liver Disease, National Sun YaD-1sen University, Kaohsiung, 80424, Taiwan

^g^Doctoral Program in Translational Medicine, National Chung Hsing University, Taichung, Taiwan.

* To whom correspondence should be addressed.

Tel: +886-4-22840485 #270

Email: i90221141ster@gmail.com; emersontseng@dragon.nchu.edu.tw

**Supporting information**

**Figures**

**
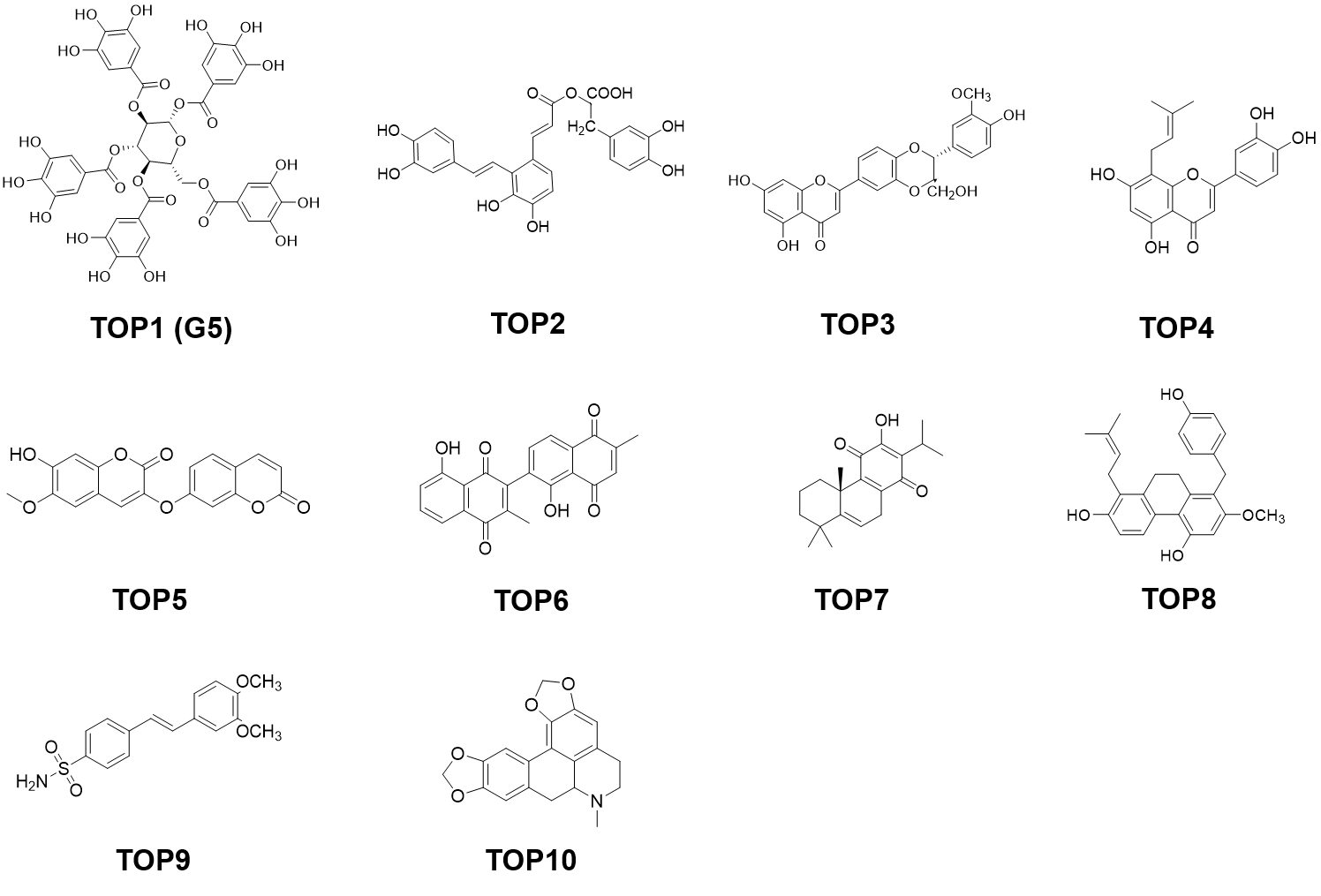
**

**Figure S1.** **The chemical structures of top 10 ranked hits identified from TCM database.**

**
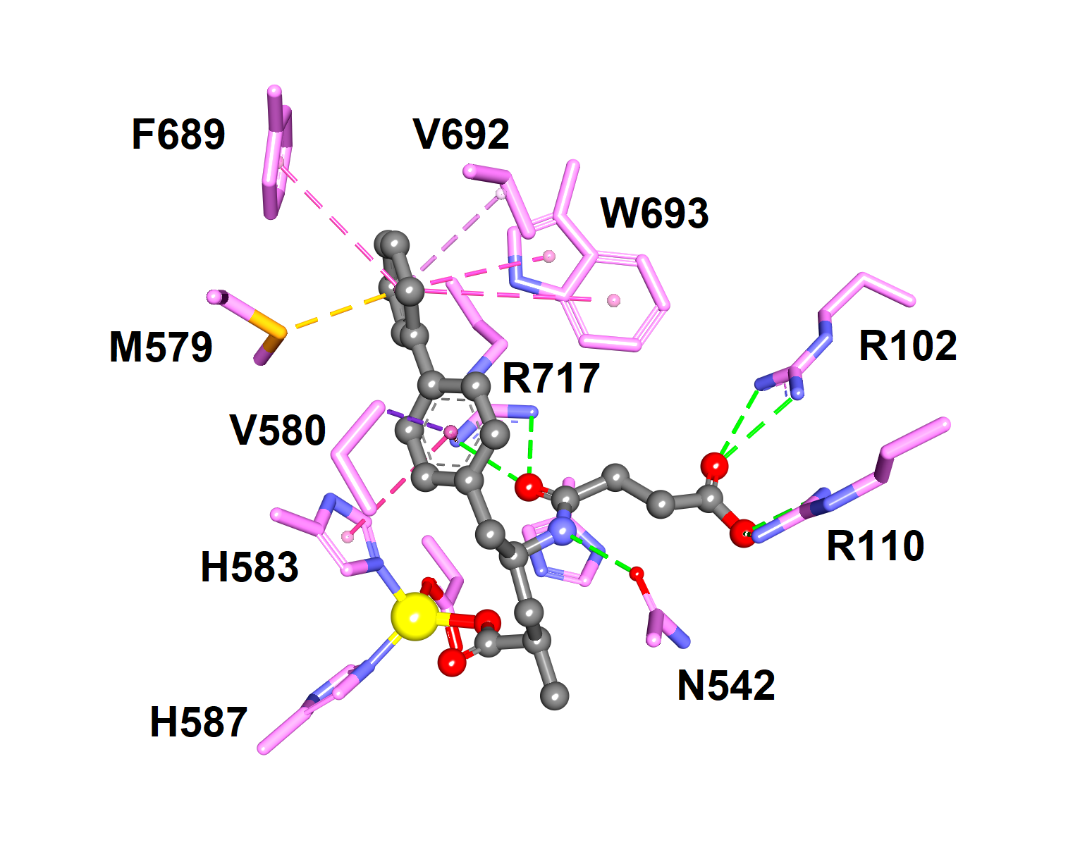
**

**Figure S2.** **Molecular interactions between human neprilysin (NEP) and sacubitrilat.** Sacubitrilat is shown in ball-and-stick representation (gray), while key NEP residues are depicted as sticks. Hydrogen bonds are indicated by green dashed lines, hydrophobic interactions by magenta dashed lines, and π–sulfur interactions by yellow dashed lines. Cation–π interactions involving R717 are also observed. Residues contributing to ligand stabilization include R102, R110, N542, R717, V580, H583, F689, V692, and W693. Coordination with the catalytic Zn²⁺ ion (yellow sphere), mediated by H583 and H587, further anchors sacubitrilat within the NEP active site, highlighting key interaction hotspots characteristic of known human NEP inhibitors.
